# Pancreatic beta cell autophagy is impaired in type 1 diabetes

**DOI:** 10.1007/s00125-021-05387-6

**Published:** 2021-01-30

**Authors:** Charanya Muralidharan, Abass M. Conteh, Michelle R. Marasco, Justin J. Crowder, Jeroen Kuipers, Pascal de Boer, Amelia K. Linnemann

**Affiliations:** 1 Department of Biochemistry and Molecular Biology, Indiana University School of Medicine, Indianapolis, IN USA; 2 Department of Pediatrics, Indiana University School of Medicine, Indianapolis, IN USA; 3 Department of Biomedical Sciences of Cells and Systems, University of Groningen, University Medical Center Groningen, Groningen, the Netherlands; 4 Center for Diabetes and Metabolic Diseases, Indiana University School of Medicine, Indianapolis, IN USA

**Keywords:** Autoantibody-positive, Autophagosome, Autophagy, Crinophagy, Lysosome, Type 1 diabetes

## Abstract

**Aims/hypothesis:**

Pancreatic beta cells are subjected to exogenous damaging factors such as proinflammatory cytokines or excess glucose that can cause accumulation of damage-inducing reactive oxygen species during the pathogenesis of diabetes. We and others have shown that beta cell autophagy can reduce reactive oxygen species to protect against apoptosis. While impaired islet autophagy has been demonstrated in human type 2 diabetes, it is unknown if islet autophagy is perturbed in the pathogenesis of type 1 diabetes. We hypothesised that beta cell autophagy is dysfunctional in type 1 diabetes, and that there is a progressive loss during early diabetes development.

**Methods:**

Pancreases were collected from chloroquine-injected and non-injected non-obese diabetes-resistant (NOR) and non-obese diabetic (NOD) mice. Age- and BMI-matched pancreas tissue sections from human organ donors (*N* = 34) were obtained from the Network for Pancreatic Organ Donors with Diabetes (nPOD). Tissue sections were stained with antibodies against proinsulin or insulin (beta cell markers), microtubule-associated protein 1 light chain 3 A/B (LC3A/B; autophagosome marker), lysosomal-associated membrane protein 1 (LAMP1; lysosome marker) and p62 (autophagy adaptor). Images collected on a scanning laser confocal microscope were analysed with CellProfiler and ImageJ. Secondary lysosomes and telolysosomes were assessed in electron micrographs of human pancreatic tissue sections (*n* = 12), and energy dispersive x-ray analysis was performed to assess distribution of elements (*n* = 5).

**Results:**

We observed increased autophagosome numbers in islets of diabetic NOD mice (*p* = 0.008) and increased p62 in islets of both non-diabetic and diabetic NOD mice (*p* < 0.001) vs NOR mice. There was also a reduction in LC3–LAMP1 colocalisation in islets of diabetic NOD mice compared with both non-diabetic NOD (*p* < 0.001) and NOR mice (*p* < 0.001). Chloroquine elicited accumulation of autophagosomes in the islets of NOR (*p* = 0.003) and non-diabetic NOD mice (*p* < 0.001), but not in islets of diabetic NOD mice; and stimulated accumulation of p62 in NOR (*p* < 0.001), but not in NOD mice. We observed reduced LC3–LAMP1 colocalisation (*p* < 0.001) in residual beta cells of human donors with type 1 diabetes vs non-diabetic participants. We also observed reduced colocalisation of proinsulin with LAMP1 in donors with type 1 diabetes (*p* < 0.001). Electron microscopy also revealed accumulation of telolysosomes with nitrogen-dense rings in beta cells of autoantibody-positive donors (*p* = 0.002).

**Conclusions/interpretation:**

We provide evidence of islet macroautophagy/crinophagy impairment in human type 1 diabetes. We also document accumulation of telolysosomes with peripheral nitrogen in beta cells of autoantibody-positive donors, demonstrating altered lysosome content that may be associated with lysosome dysfunction before clinical hyperglycaemia. Similar macroautophagy impairments are present in the NOD mouse model of type 1 diabetes.

**Graphical abstract:**

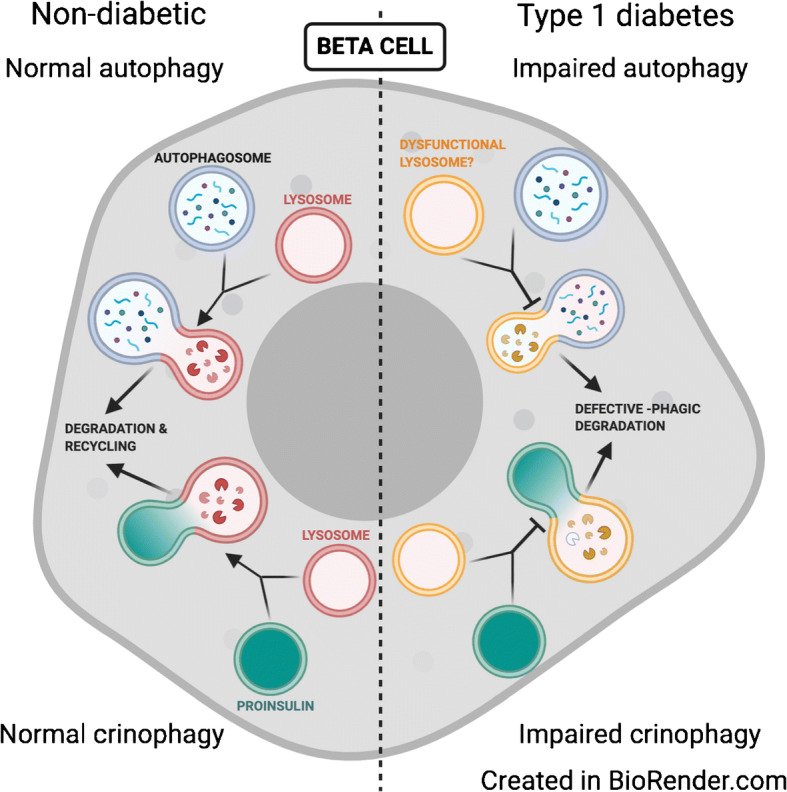

**Supplementary Information:**

The online version contains peer-reviewed but unedited supplementary material available at 10.1007/s00125-021-05387-6.

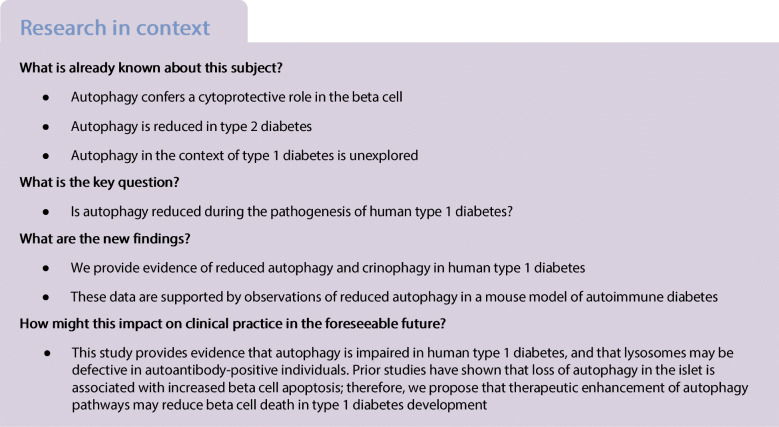



## Introduction

Beta cell reactive oxygen species (ROS) accumulation is implicated as a triggering event during the development of type 1 diabetes [1]. ROS can be generated from multiple intracellular sources, such as mitochondrial oxidative phosphorylation, metabolism of long-chain fatty acids in the peroxisome and enzyme catalysis [2]. Although low levels of ROS can promote important signalling events in the beta cell such as insulin secretion [3] and proliferation [4], excess ROS can damage cellular proteins and organelles and overwhelm endogenous mechanisms that maintain homeostasis [5].

Autophagy is an endogenous mechanism to reduce ROS and promote beta cell survival [6]. Known types of autophagy, including macroautophagy, chaperone-mediated autophagy, microautophagy and crinophagy, function as important housekeeping catabolic processes to facilitate recycling of excess or damaged cellular components and promote cellular homeostasis [7]. Macroautophagy (herein, autophagy) is a dynamic process involving a cascade of regulated events leading to engulfment of damaged proteins or organelles into double-membraned autophagosomes containing microtubule-associated protein 1 light chain 3 (MAP1LC3, or LC3), a widely used marker for mature autophagosomes. Mature autophagosomes fuse with acidic lysosomes for cargo degradation and recycling [8]. Crinophagy is a specialised form of ‘-phagic’ degradation that occurs in secretory cells, where the secretory granules fuse directly with the lysosome [9]. Although the mechanism of crinophagy is not well understood, this process plays a key role in the regulation of insulin granules [10].

The critical importance of autophagy for beta cell homeostasis and survival in the context of type 2 diabetes has been well documented [11]. Although there is no literature demonstrating impaired autophagy in the context of human type 1 diabetes, dysfunctional autophagy has been implicated in the pathogenesis of several autoimmune disorders [12], suggesting a potential role in type 1 diabetes pathogenesis. Indeed, a role for the autophagic pathway in type 1 diabetes-associated autoimmunity has been hypothesised [13]. We thus aimed to assess if there is a decline in islet autophagy associated with type 1 diabetes.

## Methods

### Mice

Non-obese diabetes-resistant (NOR) and non-obese diabetic (NOD) mice were purchased from The Jackson Laboratory (ME, USA) at ~7 weeks of age. Mice were housed in a temperature-controlled facility with a 12 h light/12 h dark cycle and were given free access to food and water. All experiments were approved by the Indiana University School of Medicine Institutional Animal Care and Use Committee. Random-fed blood glucose for NOR mice and NOD mice was monitored bi-weekly with an AlphaTrak2 glucometer (Zoetis, NJ, USA), and NOD mice were characterised as diabetic after 2 consecutive days of blood glucose readings >13.9 mmol/l. Mice were euthanised by cervical dislocation and pancreases were collected. Harvested tissues were fixed in 3.7% paraformaldehyde (vol./vol.) for 4–5 h at room temperature with gentle agitation and then transferred to 70% ethanol. Pancreases were then paraffin embedded and sectioned in the Histology Core of the Indiana Center for Musculoskeletal Health, Indiana University School of Medicine. For all experiments, female mice aged 11–26 weeks were used.

### Chloroquine injections

To analyse dynamic autophagic flux, a subset of NOR mice (11 weeks; *n* = 5), non-diabetic NOD mice (14 weeks; *n* = 5) and diabetic NOD mice (14–26 weeks; *n* = 3) were intra-peritoneally injected with 50 mg/kg of chloroquine diphosphate (Tocris Bioscience #4109, Bristol, UK). At 2 h post injection, pancreases were collected, paraffin embedded and sectioned. For non-injected controls, a subset of NOR mice (*n* = 5), non-diabetic NOD mice (*n* = 6) and diabetic NOD mice (*n* = 7) were used.

### Human organ donor characteristics

**Fig. 1:**
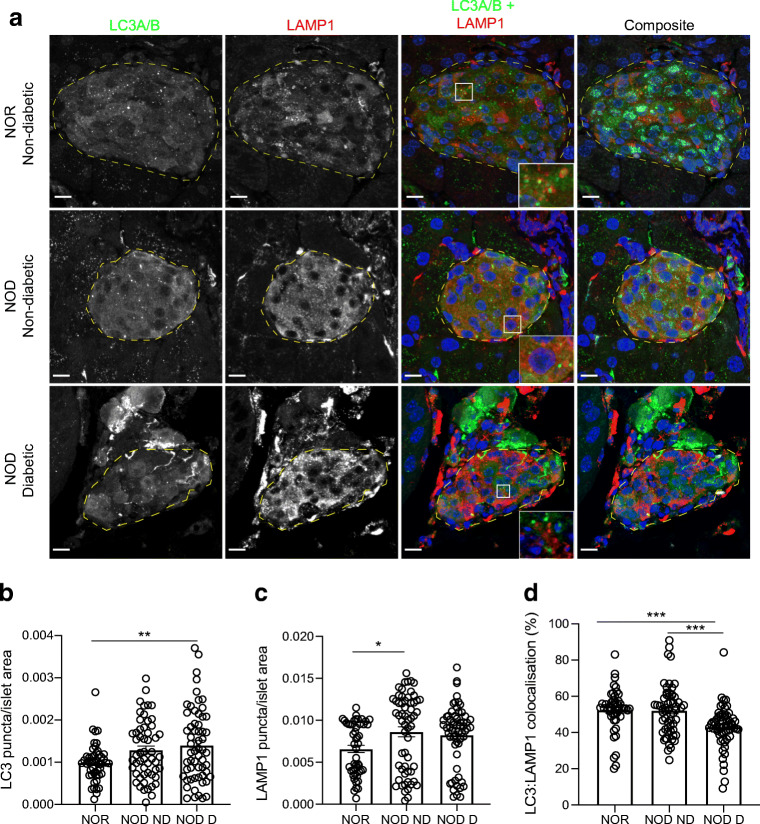
Reduced autophagy in the pancreatic islets of diabetic NOD mice. (**a**) Representative images showing immunofluorescent staining of islets in pancreas tissue sections from non-diabetic NOR, non-diabetic NOD and diabetic NOD mice. Autophagosomes (LC3A/B) are shown in green, lysosomes (LAMP1) in red, proinsulin in cyan and nuclei (DAPI) in blue. Scale bars, 10 μm. Insets show higher magnification of overlapping puncta. (**b**) Quantification of autophagosomes. (**c**) Quantification of lysosomes. (**d**) Quantification of colocalisation of autophagosomes with lysosomes in islet. Each circle denotes an islet. *p<0.05; **p<0.01; ***p<0.001 (one-way ANOVA). D, diabetic; ND, non-diabetic

We obtained deidentified pancreatic tissue sections from 34 human organ donors through the JDRF Network for Pancreatic Organ Donors with Diabetes (nPOD; Table 1). Because samples were deidentified, they were exempt from institutional review board oversight. Samples included sections from 12 non-diabetic control donors (six male and six female donors), 12 autoantibody-positive donors (six male, six female) and ten donors with type 1 diabetes (five male, five female) that had residual insulin-positive islets. Age- (26.15 ± 2 years) and BMI- (26.55 ± 1 kg/m^2^) matched donor samples were used. A breakdown of age and BMI in each group is shown in electronic supplementary material (ESM) Fig.1 and in the Human Islet Checklist. The duration of diabetes ranged from <1 year to 32.5 years.

**Table 1:** nPOD donor characteristics

Group	Donor ID	Age (years)	Sex	BMI (kg/m^2^)	C-peptide (nmol/l)	T1D duration (years)	Autoantibody
Non-diabetic	6015	39	F	32.2	0.66	–	–
Non-diabetic	6160	22.1	M	23.9	0.13	–	–
Non-diabetic	6162	22.7	M	28.9	2.54	–	–
Non-diabetic	6178	24.5	F	27.5	1.52	–	–
Non-diabetic	6235	30	M	25.4	2.70	–	–
Non-diabetic	6335	18.8	M	23.6	2.95	–	–
Non-diabetic	6339	23.3	M	25	3.52	–	–
Non-diabetic	6373	15.7	M	25	4.37	–	–
Non-diabetic	6034	32	F	25.2	1.05	–	–
Non-diabetic	6234	20	F	25.6	2.30	–	–
Non-diabetic	6331	27.1	F	24	1.00	–	–
Non-diabetic	6333	27.1	F	24.9	3.12	–	–
Non-diabetic	6226	38	F	27.2	1.29	–	–
Non-diabetic	6227	17	F	26.4	0.92	–	–
Non-diabetic	6229	31	F	26.9	2.08	–	–
Non-diabetic	6230	16	M	19.4	1.74	–	–
Non-diabetic	6232	14	F	20.83	6.50	–	–
Aab+	6147	23.8	F	32.9	1.06	–	GADA
Aab+	6167	37	M	26.3	1.81	–	IA2A, ZnT8A
Aab+	6151	30	M	24.2	1.83	–	GADA
Aab+	6170	34.5	F	36.9	1.43	–	GADA
Aab+	6181	31.9	M	21.9	0.02	–	GADA
Aab+	6301	26	M	32.1	1.31	–	GADA
Aab+	6310	28	F	22.4	3.51	–	GADA
Aab+	6314	21	M	23.8	0.50	–	GADA
Aab+	6397	21.16	F	29.6	4.26	–	GADA
Aab+	6400	25.15	M	22.2	1.39	–	GADA
Aab+	6450	22	F	24.4	1.82	–	GADA, ZnT8A
Aab+	6483	30.46	F	20.8	0.74	–	GADA
Aab+	6388	25.2	F	26	0.46	–	GADA, mIAA
Aab+	6303	22	M	31.9	1.01	–	GADA
Aab+	6197	22	M	28.2	5.83	–	GADA, IA2A
Aab+	6156	40	M	19.8	4.45	–	GADA
T1D	6046	18.8	F	25.2	0.02	8	IA2A, ZnT8A
T1D	6302	38.5	M	20.5	0.06	32.5	–
T1D	6306	19	M	24.5	0.01	5	mIAA
T1D	6325	20	F	31.2	0.05	6	GADA, IA2A, mIAA
T1D	6328	39	M	24	0.01	20	GADA, mIAA
T1D	6367	24	M	25.7	0.13	2	–
T1D	6405	29.1	F	42.5	0.61	0.6	GADA, IA2A, ZnT8A
T1D	6414	23.1	M	28.4	0.05	0.43	GADA, mIAA, ZnT8A
T1D	6435	24.75	F	26.9	0.03	14.75	–
T1D	6477	19.87	F	25.3	0.06	8	GADA, IA2A, mIAA

ID, identity; T1D, type 1 diabetes; F, female; M, male; Aab+, autoantibody-positive; GADA, GAD autoantibodies; IA2A, insulinoma-associated protein 2 autoantibodies; ZnT8A, zinc transporter 8 autoantibodies; mIAA, micro-insulin autoantibodies

### Immunofluorescence analysis

Tissue sections were dewaxed in xylene and hydrated in serial dilutions of ethanol (2×; 100%, 90% and 70%) followed by water. Heat- and citrate-based antigen retrieval was performed using Vector Laboratories (CA, USA) antigen unmasking solution (H-3300) for 20 min and then the slides were allowed to cool to room temperature for 1 h. Slides were blocked with Dako blocking buffer (Agilent Technologies, CA, USA, #X0909) prior to incubation with antibodies in Dako antibody diluent (Agilent Technologies #S3022). Antibodies and the corresponding dilutions used are listed as follows: Developmental Studies Hybridoma Bank (DSHB, Iowa, USA) mouse anti-Proinsulin (GS-9A8; 1:50), rat anti-lysosomal-associated membrane protein 1 (LAMP1) (DSHB 1D4B; 1:50), rabbit anti-LC3A/B (Cell Signaling Technology, MA, USA, #12741; 1:100), rabbit anti-p62 (Abcam, Cambridge, UK, #ab91526; 1:200) and guinea pig anti-insulin (BioRad, CA, USA, #5330-0104G; 1:500). Highly cross-adsorbed fluorescently conjugated secondary antibodies were used. Images were collected on either a Zeiss (Carl Zeiss, Oberkochen, Germany) LSM 700 confocal microscope using a 63X/1.4 numerical aperture oil objective, or a Zeiss LSM 800 confocal microscope equipped with Airyscan using a 63X/1.4 numerical aperture oil objective.

### CellProfiler analysis flow of logic

Fluorescence intensity measurements, puncta counts and colocalisation analyses were automated using CellProfiler (cellprofiler.org) versions 3.1.5/8/9 [14]. Background from each image was first subtracted by removing the lower-quartile intensity across each channel. Regions of interest (ROIs) for human pancreatic tissues were defined by proinsulin-positive area and ROIs for mouse pancreatic tissues were defined by manually identifying the islet area. Puncta for LC3, LAMP1, p62 and proinsulin were identified by discarding objects outside a pixel diameter range, after applying median filtering (ESM Fig. 2). For human islets, the puncta count was normalised to the proinsulin-positive area. For mouse islets, the puncta count was normalised to the area of the islet ROI. For colocalisation analysis, parent and child objects were defined. For example, for LC3 puncta colocalisation analysis with LAMP1 puncta, LAMP1 and LC3 were defined as parent and child objects, respectively, and the percentage of LC3 puncta co-compartmentalising with LAMP1 puncta was determined by following the CellProfiler tutorial for object overlap-based colocalisation [15].

### Human electron microscopy data analysis

Electron microscopy (EM) images from control and autoantibody-positive nPOD donor pancreatic tissue sections were analysed from the Nanotomy repository ([16]; Table 1). At least 48 beta cells were analysed per group. Structures that morphologically resembled lipofuscin bodies, as described by Cnop et al [17], were quantified within beta cells. Lipofuscin bodies are also named residual bodies, tertiary lysosomes and, more recently, telolysosomes. Elemental analysis of nitrogen, osmium and phosphorus for telolysosomes and secondary lysosomes in non-diabetic (*n* = 2) and autoantibody-positive (*n* = 3) donor sections was performed using EM energy dispersive x-ray analysis (EDX) as described previously [18] on cells that could be identified with certainty as beta cells by the presence of insulin granules.

### Statistical analysis

**Fig. 2:**
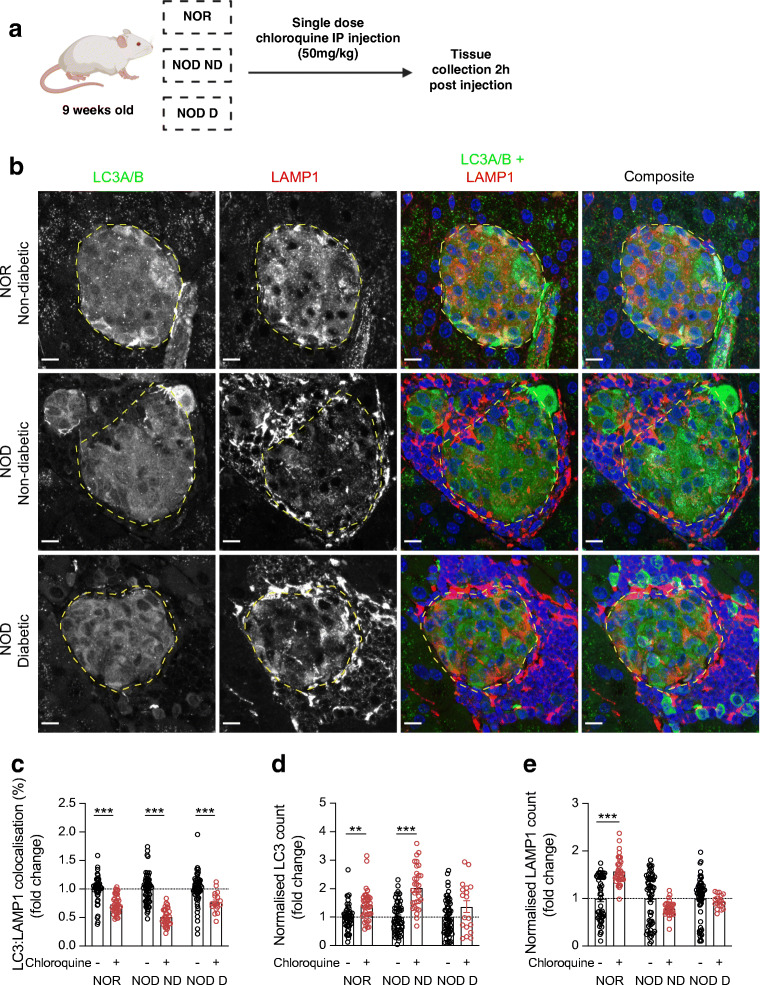
Impaired autophagic flux in the islets of diabetic NOD mice*.* (**a**) Schematic of autophagic flux assessment experiment, created in BioRender.com. (**b**) Representative images showing immunofluorescent staining of islets of pancreas tissue sections from chloroquine-injected non-diabetic NOR and non-diabetic NOD and diabetic NOD mice. Autophagosomes (LC3A/B) are shown in green, proinsulin in cyan, lysosomes (LAMP1) in red and nuclei (DAPI) in blue. Scale bars, 10 μm. (**c**) Quantification of colocalisation of autophagosomes with lysosomes in islets. (**d**) Quantification of autophagosomes. (**e**) Quantification of lysosomes. Each circle denotes an islet. **p<0.01; ***p<0.001 (two-way ANOVA with multiple comparisons). D, diabetic; ND, non-diabetic

**Fig. 3:**
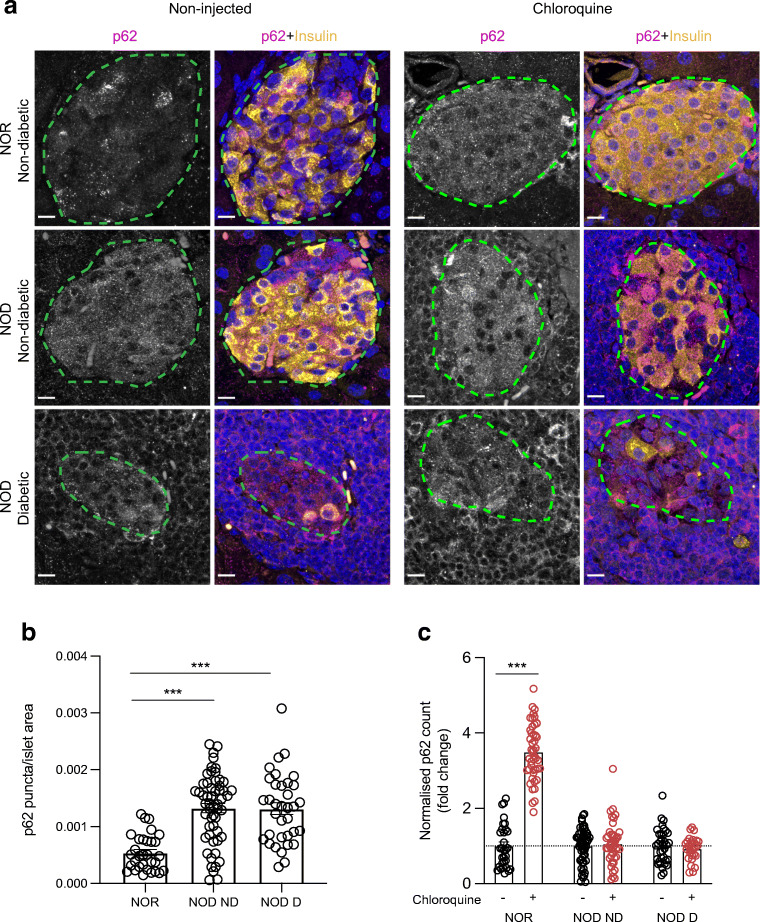
Impaired islet p62 degradation in non-diabetic and diabetic NOD mice. (**a**) Representative images showing immunofluorescent staining of islets of chloroquine-injected NOR and NOD mouse pancreatic tissue sections. p62 in magenta, insulin in yellow and nuclei (DAPI) in blue. Scale bars, 10 μm. (**b**) Quantification of p62 puncta in islet area in non-injected animals. (**c**) Fold change comparison of p62 puncta in islet area of chloroquine-injected animals. Each circle denotes an islet. ***p<0.001 (two-way ANOVA with multiple comparisons). D, diabetic; ND, non-diabetic

**Fig. 4:**
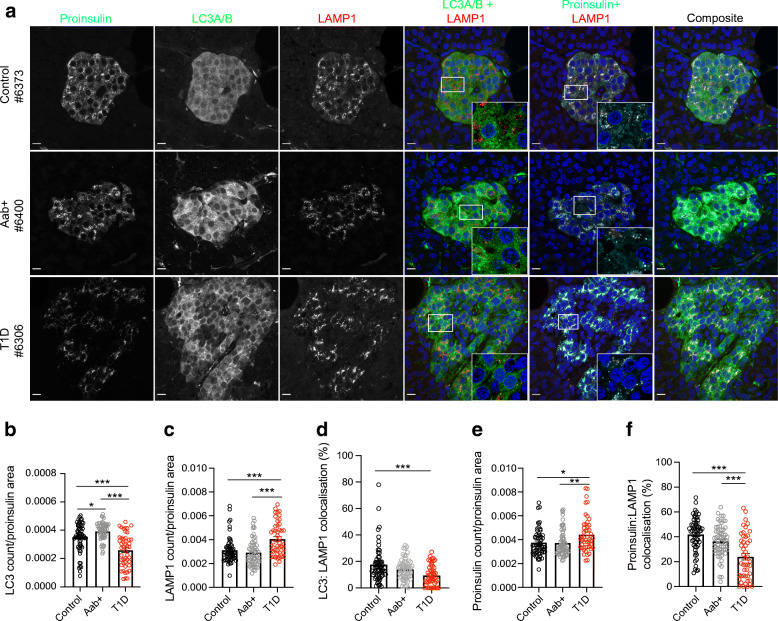
Reduced autophagy and crinophagy in the pancreatic beta cells of human donors with type 1 diabetes. (**a**) Representative images showing immunofluorescent staining of islets in proinsulin-positive cells (cyan) of pancreas tissue sections from non-diabetic, autoantibody-positive (Aab+) and type 1 diabetic organ donors. Autophagosomes (LC3A/B) are shown in green, lysosomes (LAMP1) in red and nuclei (DAPI) in blue. Scale bars, 10 μm. Insets show higher magnification of overlapping puncta collected by Airyscan imaging at 63× with a 2.5× zoom factor. (**b**) Quantification of lysosomes. (**c**) Quantification of autophagosomes. (**d**) Quantification of colocalisation of autophagosomes with lysosomes in proinsulin-positive cells. (**e**) Quantification of proinsulin puncta. (**f**) Quantification of colocalisation of proinsulin with lysosomes in proinsulin-positive cells. Each circle denotes an islet. *p<0.05; **p<0.01; ***p<0.001 (one-way ANOVA with multiple comparisons). T1D, type 1 diabetes

**Fig. 5:**
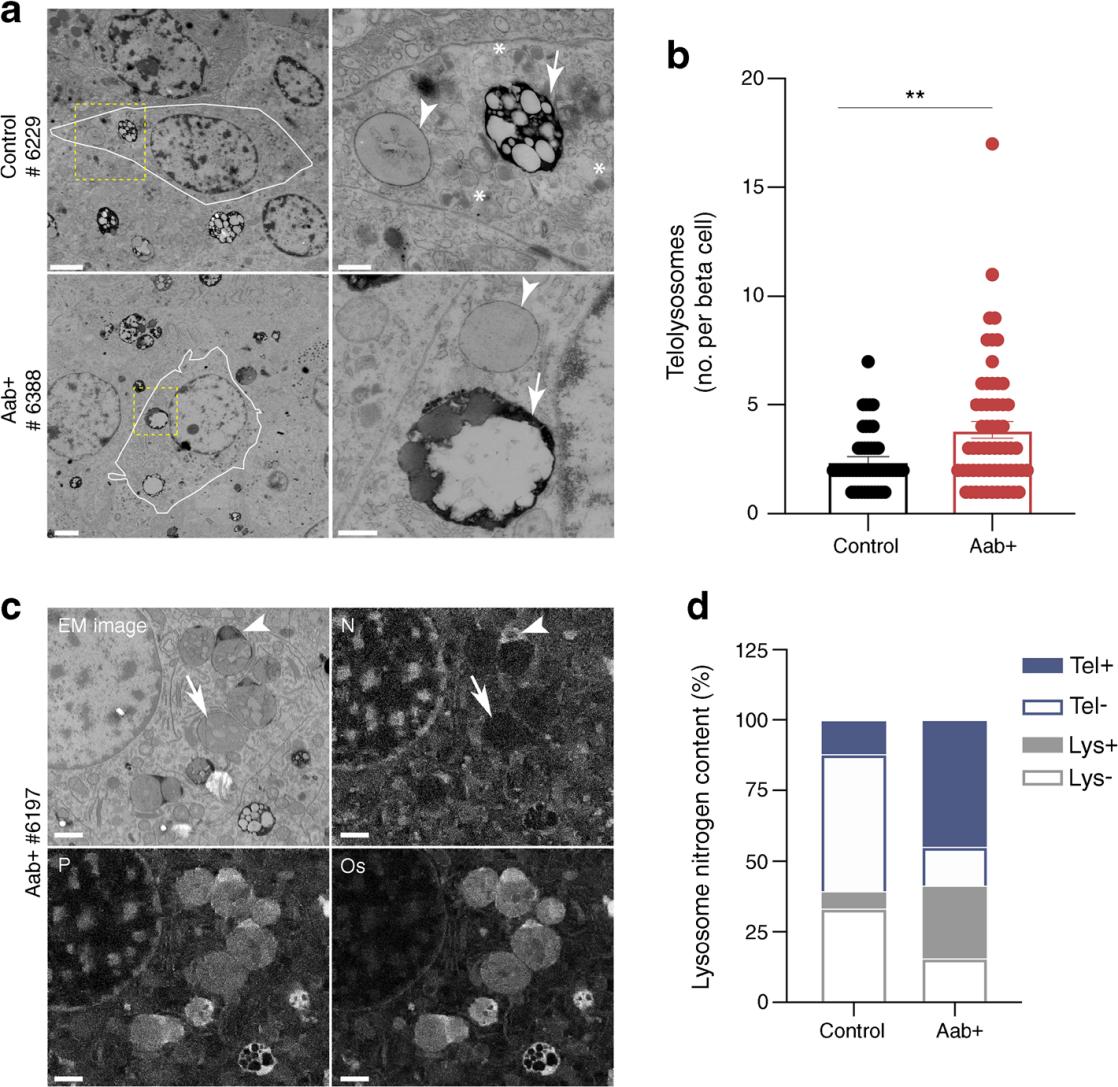
Increased presence of telolysosomes in beta cells of autoantibody-positive human donors. (**a**) Representative images showing electron microscopic images of beta cells of non-diabetic and autoantibody-positive (Aab+) organ donors. Left panels: scale bars, 2 μm. Right panels represent zoomed-in regions: scale bars, 500 nm. Arrow heads indicate secondary lysosomes, arrows with solid lines indicate telolysosomes and arrows with dashed lines indicate insulin granules. (**b**) Quantification of telolysosomes. Each circle denotes an islet. (**c**) Representative images showing EDX analysis of elements: nitrogen (N), phosphorus (P) and osmium (Os). Arrowheads indicate telolysosomes with nitrogen ring in the rim; arrows indicate telolysosomes without nitrogen rim. Scale bars, 1 μm (**d**) Quantification of percentage occupied by secondary lysosomes without nitrogen rim (Lys−), secondary lysosomes with nitrogen rim (Lys+), telolysosomes without nitrogen rim (Tel−) and telolysosomes with nitrogen rim (Tel+). **p<0.01 (unpaired student *t* test)

At least 47 proinsulin-positive islets were analysed from the pancreatic tail region for each donor group and at least 18 islets were analysed for each mouse group, with representative images shown (Figs 1, 2, 3 and 4). For EM image analysis, at least 48 insulin-positive beta cells were analysed from the pancreatic head or body region for each donor group, with representative images shown in Fig. 5. Data analysed using CellProfiler were compared between groups by one-way ANOVA with multiple comparisons (Tukey’s post hoc test), two-way ANOVA with multiple comparisons (Sidak’s post hoc test) or unpaired *t* test, as deemed appropriate, using GraphPad Prism v8.0, and are represented as mean ± SEM. *p* values <0.05 were considered statistically significant.

## Results

### Evidence of impaired autophagy in diabetic NOD mice

To determine if autophagy is impaired in a mouse model of spontaneous autoimmune diabetes, we analysed pancreases from diabetic NOD mice, comparing them with both non-diabetic NOD mice and NOR mice, which do not develop insulitis or diabetes and are MHC-matched to NOD mice [19]. Representative immunofluorescent images are shown in Fig. 1a. We observed a significant increase in LC3 puncta (autophagosome marker) in diabetic NOD mouse islets (*p* = 0.008) and a nonsignificant increase in autophagosomes in non-diabetic NOD islets (*p* = 0.083) when compared with islets of NOR mice (Fig. 1b). This was accompanied by a significant increase in LAMP1 puncta (lysosome marker) in non-diabetic NOD mice (*p* = 0.019) and a nonsignificant increase in diabetic NOD mice (*p* = 0.067) vs NOR mice (Fig. 1c).

The final step of autophagy is the degradation of autophagosomes and their cargo by lysosomal acid hydrolases. Therefore, we reasoned that if autophagy is impaired in the context of spontaneous autoimmune diabetes, this would be evident through altered colocalisation of LC3 with LAMP1 (autophagosome and lysosome markers, respectively). To assess this, we quantified the percentage of LC3 colocalised with LAMP1. We observed a significant reduction in the LC3–LAMP1 colocalisation in diabetic NOD mouse islets when compared with either NOR or non-diabetic NOD mouse islets (*p* < 0.001 for both; Fig. 1d). There was no significant difference in the colocalisation of LC3 with LAMP1 when comparing NOR with non-diabetic NOD mouse islets (Fig. 1d). Collectively, these data suggest an impairment in macroautophagy in the islets of diabetic NOD mice.

### Autophagic flux is impaired in diabetic NOD mice

Although we observed a reduction in the percentage of autophagosomes fusing with lysosomes in diabetic NOD mouse islets, these data do not necessarily capture the dynamic nature of the autophagic degradation process. Therefore, to assess autophagic flux and characterise basal autophagy status in NOD mice, we injected NOR mice and NOD mice (non-diabetic and diabetic) with chloroquine, a lysomotrophic drug that impairs the fusion of autophagosomes with lysosomes [20], thus halting the degradation of formed autophagosomes. Our experimental approach is shown in Fig. 2a, and representative immunofluorescent images of pancreases from chloroquine-injected mice are shown in Fig. 2b. To ensure that chloroquine functioned as intended and to validate our colocalisation analysis, we compared the islets of non-injected and chloroquine-injected NOR and NOD (non-diabetic and diabetic) mice. We observed a statistically significant reduction in the colocalisation of LC3 with LAMP1 in islets of chloroquine-injected mice when compared with non-injected animals across all three groups (Fig. 2c), suggesting that autophagosome:lysosome fusion was indeed disrupted by the chloroquine. We also observed a significant increase in islet LC3 puncta in chloroquine-injected NOR (*p* = 0.003) and non-diabetic NOD mice (*p* < 0.001) compared with their respective non-injected controls (Fig. 2d), suggesting effective blocking of degradation by chloroquine injections in NOR and non-diabetic NOD mice. In contrast, there was no difference in islet LC3 puncta count in chloroquine-injected diabetic NOD mice vs non-injected controls (Fig. 2d), suggesting an impairment of autophagic flux in these mice. Comparison of islet LC3 puncta between chloroquine-injected non-diabetic NOD and chloroquine-injected diabetic NOD mice revealed a significant decrease in the number of chloroquine-induced accumulating puncta in the context of diabetes (*p* = 0.028). Additionally, we observed a significant increase in islet lysosome numbers in chloroquine-injected NOR mice (*p* < 0.001; Fig. 2e). However, there was no difference in lysosomes of chloroquine-injected NOD mice vs their corresponding non-injected controls, regardless of diabetes status (Fig. 2e).

Next, we analysed p62 in islets of NOR and NOD mice (Fig. 3a). p62 is an autophagy adaptor protein that binds to both LC3 and other ubiquitinated proteins being targeted for degradation to aid in bringing them together [21], and is typically degraded within the lysosome during the autophagic degradation process [22]. Therefore, one would expect a reduction in p62 if autophagic flux is intact [23, 24]. At baseline, we observed p62 accumulation in islets of both non-diabetic and diabetic NOD mice (*p* < 0.001 in both cases) when compared with NOR mice (Fig. 3b). Chloroquine injection led to a significant increase in p62 levels in islets of NOR mice (*p* < 0.001), whereas there was no change elicited by chloroquine in NOD mice, regardless of diabetes status (Fig. 3c). These data collectively suggest an impairment in autophagic flux in the islets of NOD mice that is more pronounced in the residual islets of diabetic NOD mice. Our analyses are also suggestive of impairment in the final stage of the autophagic degradation process in the context of spontaneous autoimmune diabetes in mice.

### Autophagosome numbers are reduced in beta cells of human type 1 diabetes donors

Impairment in autophagy has previously been demonstrated in the context of human type 2 diabetes [25, 26], but not in human type 1 diabetes. Therefore, we obtained human pancreases through nPOD. We first assessed the LC3 (autophagosome) puncta and LAMP1 (lysosome) puncta within the proinsulin-positive area of non-diabetic control, autoantibody-positive or type 1 diabetic organ donor pancreases (Fig. 4a). We observed a significant reduction of autophagosomes in beta cells of donors with type 1 diabetes when compared with both non-diabetic and autoantibody-positive donors (*p* < 0.001; Fig. 4b). Interestingly, we also observed increased autophagosome numbers in the beta cells of autoantibody-positive donors when compared with beta cells of non-diabetic donors (*p* = 0.046; Fig. 4b). These data suggest accumulation of autophagosomes prior to the onset of clinical hyperglycaemia. Similarly, lysosome numbers were significantly increased in the beta cells of donors with type 1 diabetes when compared with non-diabetic and autoantibody-positive donors, respectively (*p* < 0.001 for both; Fig. 4c).

### Autophagy is impaired in human type 1 diabetes

We next quantified colocalisation of LC3 (autophagosome) puncta with LAMP1 (lysosome) puncta in the proinsulin-positive islet cells of non-diabetic, autoantibody-positive and type 1 diabetic human organ donors (Fig. 4d). We did not observe a robust difference in LC3–LAMP1 colocalisation between non-diabetic and autoantibody-positive individuals. However, there was a significant reduction in colocalisation of LC3 with LAMP1 in the residual beta cells of type 1 diabetic donors vs non-diabetic donors (*p* < 0.001), and a nonsignificant decrease of colocalisation in the beta cells of type 1 diabetic donors vs autoantibody-positive donors (*p* = 0.067; Fig. 4d). This suggests that the final stages of autophagic degradation are impaired in the context of type 1 diabetes. Additionally, there was no correlation between percentage of LC3–LAMP1 colocalisation and disease duration or C-peptide levels (ESM Fig. 3).

### Crinophagy is reduced in human type 1 diabetes

Crinophagy is another method of ‘-phagic’ degradation by which the secretory granule directly fuses with the lysosomes. Crinophagy has been implicated in the presentation of altered peptides that recruit T cells to the beta cell [27]. To determine if there is alteration of crinophagy in type 1 diabetes, we quantified the amount of proinsulin (Fig. 4e), and also the percentage of proinsulin that colocalised with lysosomes (Fig. 4f). We observed a significant increase in proinsulin (*p* = 0.011, *p* = 0.009) but a reduction in proinsulin–lysosome colocalisation in beta cells of donors with type 1 diabetes (*p* < 0.001, *p* < 0.001) when compared with beta cells of both non-diabetic and autoantibody-positive donors. There was no correlation between crinophagy and disease duration or C-peptide levels (ESM Fig. 3). These data support the conclusion that crinophagy is also reduced in the context of type 1 diabetes.

### Telolysosomes are increased in autoantibody-positive individuals

Autophagy and crinophagy are dependent on several factors, including the integrity of the lysosome acidification machinery to create an acidic environment that is capable of protein and organelle degradation. Telolysosomes (also known as lipofuscin bodies, residual bodies or tertiary lysosomes) are lysosomes that contain undigestible biological garbage or cellular components such as highly oxidised, covalently crosslinked proteins, sugars or lipids [28]. Telolysosomes have been shown to accumulate within post-mitotic cells that are long-lived and are positively correlated with ageing in the beta cell [29]. Additionally, oxidative stress and inhibition of lysosomal enzymes have been associated with increased accumulation of telolysosomes [30]. Therefore, we took advantage of electron microscopic images of nPOD pancreas tissue in the Nanotomy repository [16], available from www.nanotomy.org/OA/nPOD. We identified telolysosomes (lipofuscin bodies) as described [17] (Fig. 5a) and quantified them in a series of samples from non-diabetic and autoantibody-positive organ donors with some of the samples that were also analysed by immunofluorescence analysis (Fig. 5b). We observed a significant increase in the number of telolysosomes in beta cells of autoantibody-positive individuals when compared with non-diabetic control individuals (*p* = 0.002). This suggests that autophagy may be impaired early in type 1 diabetes pathogenesis prior to the development of hyperglycaemia.

### Elemental analysis of lysosomes and telolysosomes

We went on to perform elemental analysis to identify phosphorous, osmium and nitrogen in beta cell lysosomes and telolysosomes from non-diabetic and autoantibody-positive donors (Fig. 5c). We observed phosphorus colocalising with osmium used in sample preparation to fix (membrane) lipids, suggesting presence of phospholipids in all samples. We also observed an increased percentage of lysosomes/telolysosomes with nitrogen accumulation associated with the phospholipid-rich lysosome core in beta cells of autoantibody-positive donors (Fig. 5c,d; 70% of lysosomes + telolysosomes vs 18% in non-diabetic donors). Zinc and sulphur, being components of insulin, were below the detection limit in these lysosomes. The origin of the nitrogen rim remains unclear.

## Discussion

Type 1 diabetes pathogenesis is classically viewed with an immune-centric focus, due to the critical role for autoimmunity in beta cell apoptosis. However, recent evidence suggests early precipitating events may originate within the islet itself, leading to destructive beta cell targeting by the immune system [1, 31]. Autophagy is an important player in beta cell survival and growth, contributing to cellular homeostasis and stress response [32]. While the role of autophagy in the context of type 2 diabetes has been studied extensively [33, 34], its role in type 1 diabetes is relatively unexplored. Here we show that beta cell autophagosome clearance is impaired in the NOD model of diabetes, and provide data suggesting that beta cell autophagy and crinophagy are impaired in human type 1 diabetes. Therefore, we propose that impaired autophagy and/or crinophagy play a role in type 1 diabetes disease pathogenesis.

Prior studies have hinted at defective autophagy in the context of type 1 diabetes. For example, a type 1 diabetes susceptibility gene, *Clec16a*, increased rodent beta cell insulin secretion through the stimulation of a selective form of autophagy known as mitophagy, whereas humans with the susceptibility allele exhibited both reduced *CLEC16A* expression and increased HbA_1c_ [35]. Another type 1 diabetes susceptibility gene, *Cathepsin H* (*CTSH*) [36, 37], is a lysosomal cysteine protease that is crucial for lysosomal protein degradation. Importantly, the *CTSH* susceptibility allele is associated with faster disease progression in newly diagnosed diabetes and reduced beta cell function in healthy humans [38].

Our observation of reduced colocalisation of LC3 and LAMP1 in both human type 1 diabetes and NOD mice, coupled with our observations of impaired autophagic flux in diabetic NOD mice, suggests that the later stages of the autophagy degradation process, and not the formation of autophagosomes, are likely impaired. This observation raises several questions: (1) Are there defects in vesicle fusion machinery leading to decreased autophagy in type 1 diabetes? (2) Is there a defect in transport of autophagosomes to lysosomes along microtubules? (3) Are the lysosomes themselves mis-localised and not readily available for fusion? Or, (4) is there a defect in lysosome function, perhaps due to localisation or acidity [38, 39]?

Of the possibilities listed above, our data suggest that lysosome defects are associated with type 1 diabetes pathogenesis. Lysosomes are major nutrient-sensing organelles that not only function as a degradation system, but also play a major role in stress adaptation [40]. Defects in lysosomal function and/or acidity are implicated in ageing and the pathogenesis of various diseases, such as Parkinson’s, Huntington’s, Alzheimer’s, Gaucher, Niemann–Pick, mucolipidosis, Batten’s and lipofuscinosis [41, 42]. The lysosomal protease, *CTSH*, also provides a direct link between lysosome function and type 1 diabetes susceptibility, where the *CTSH* susceptibility allele is associated with impaired human beta cell function [38]. In support of the hypothesis that lysosome dysfunction may be involved in diabetes pathogenesis, genetic knockout of a key isoform of the vacuolar type H^+^ ATPase that is present on lysosomes also led to impaired mouse insulin secretion [43]. These studies implicate a crucial role for lysosomes in the regulation of beta cell homeostasis and function.

EM has long been a gold standard in autophagy studies. We analysed beta cells from nPOD donor pancreases, observing a significant increase in telolysosomes, or lipofuscin bodies, in beta cells of autoantibody-positive donors. These represent lysosomes that have accumulated oxidised and highly crosslinked proteins, lipids and sugars that are undigestible. Our data suggest that in addition to their characteristic visual features that differentiate telolysosomes from normal secondary lysosomes, many can be further differentiated by the presence of a peripheral nitrogen accumulation associated with a phospholipid-rich core. Of note, despite not being able to digest the lysosome contents, lipofuscin bodies constantly receive additional lysosomal enzymes from the cell in an attempt to digest the materials, ultimately further contributing to a relative deficiency of lysosomal enzymes in the cell [44]. Our observation of increased telolysosomes in the beta cells of autoantibody-positive individuals, taken together with our observation of increased autophagosomes in beta cells of autoantibody-positive donors, hints that impairment in autophagy could occur before the development of clinical hyperglycaemia in autoantibody-positive individuals, perhaps due to defective lysosomes. Collectively, these observations support an important role for beta cell lysosomes in the pathogenesis of type 1 diabetes.

In addition to impaired macroautophagy, we also observed evidence of reduced crinophagy in the beta cells of individuals with type 1 diabetes. It was recently demonstrated that crinophagic bodies may contain short peptides with potentially immunogenic epitopes, suggesting that altered crinophagy could lead to the presentation of altered peptides that recruit T cells to the beta cell [27]. A series of independent studies demonstrate detectable levels of proinsulin in the sera of individuals with type 1 diabetes [45], and increased levels of proinsulin secretion from autophagy-deficient cells [46]. Together, these studies in conjunction with our observations raise the possibility of a potential role for secretion of proinsulin from crinophagic bodies in type 1 diabetes. Additionally, our data suggest that, although there is not globally decreased crinophagy in early type 1 diabetes pathogenesis (i.e., autoantibody-positive donor beta cells), the processing of proteins within the crinophagic bodies is perhaps already altered, appearing as telolysosomes with undigested material by EM analysis. We therefore propose that this possibility, alongside the potential involvement of defects in other ‘-phagic’ degradation pathways such as vesicophagy [47] or pathways independent of autophagy, should be considered.

Several limitations remain that must also be addressed in future studies. For example, although our data are suggestive, in our current study we do not fully answer the question of whether impaired autophagy or crinophagy is a cause or consequence of hyperglycaemia. This holds true in the case of our NOD mouse studies as well, since the development of diabetes in the NOD mouse model is relatively stochastic and there are currently no clear-cut ways to predict which animals will become diabetic at any given point in time. Autophagy and crinophagy are dynamic processes, and our analyses represent only a static snapshot in time. Our observation of reduced number of autophagosomes, and reduced colocalisation of LC3 with LAMP1 in beta cells of donors with type 1 diabetes, suggest three possibilities: (1) reduced formation of autophagosomes; (2) increased clearance of autophagosomes; and/or (3) release of autophagosomes from the cells. However, due to the static nature of the study and the practical inability to perform flux-based experiments in human donor samples, we are unable to pinpoint exactly where the defect could be with the current approaches. However, it is clear that exogenous factors may dynamically change autophagic flux associated with lysosome dysfunction in the beta cell, as is the case for our data and evidence in the literature of the effects elicited by proinflammatory cytokines [48]. Therefore, while our data suggest globally impaired autophagy and crinophagy in human type 1 diabetes, perhaps linked to defective lysosome function, they are only suggestive and do not tell us if the kinetics are perturbed during diabetes pathogenesis. Since the data obtained from our NOD mice are concordant with our human data, NOD mice can potentially be used as a model to study type 1 diabetes-associated autophagy impairment. Future studies that can incorporate analysis of dynamics in the intact mouse pancreas, such as novel non-invasive imaging approaches [49], or intravital microscopy approaches [50] to selectively study beta cell mass, function and signalling at the single-islet and subcellular level, will be crucial to fully understanding autophagy defects in the context of type 1 diabetes pathogenesis.

### Conclusion

In conclusion, we provide evidence of impaired beta cell autophagy and crinophagy in human type 1 diabetes. These results have potential clinical implications for type 1 diabetes prevention and we anticipate that further studies of autophagy and crinophagy in the context of type 1 diabetes will yield additional insight for therapeutic targets in the future.

## Supplementary information

ESM (PDF 1006 kb)

## Data Availability

All data generated or analysed during this study are included in this published article (and its supplementary information files). Data for individual human donors, broken down by donor ID, are available from the corresponding author on reasonable request.

## Abbreviations

DSHB: Developmental Studies Hybridoma Bank
EDX: Energy dispersive x-ray analysis
EM: Electron microscopy
LAMP1: Lysosomal-associated membrane protein 1
LC3: Microtubule-associated protein 1 light chain 3
NOD: Non-obese diabetic
NOR: Non-obese diabetes-resistant
nPOD: Network for Pancreatic Organ Donors with Diabetes
ROI: Region of interest
ROS: Reactive oxygen species
