# Supplementary material for: Pancreatic beta cell autophagy is impaired in type 1 diabetes
Source: Diabetologia. 2021 Jan 30;64(4):865–77. doi: 10.1007/s00125-021-05387-6 (PMC7940272; doi:10.1007/s00125-021-05387-6)
Supplement: Supplementary file 1 — (PDF 1006 kb) [file 125_2021_5387_MOESM1_ESM.pdf]

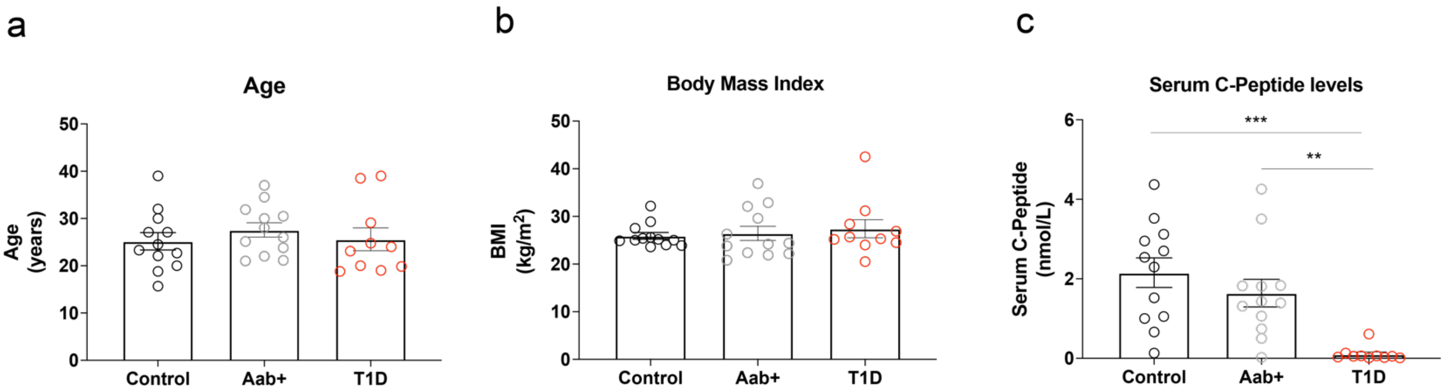

ESM Figure 1. Characteristics of pancreatic tissue sections of donors used in the immunofluorescence staining experiments of human pancreatic tissues. **(A)** Age of the donors. **(B)** Body mass index of the donors. **(C)** Serum C-peptide levels of the donors. **p<0.01; ***p<0.001

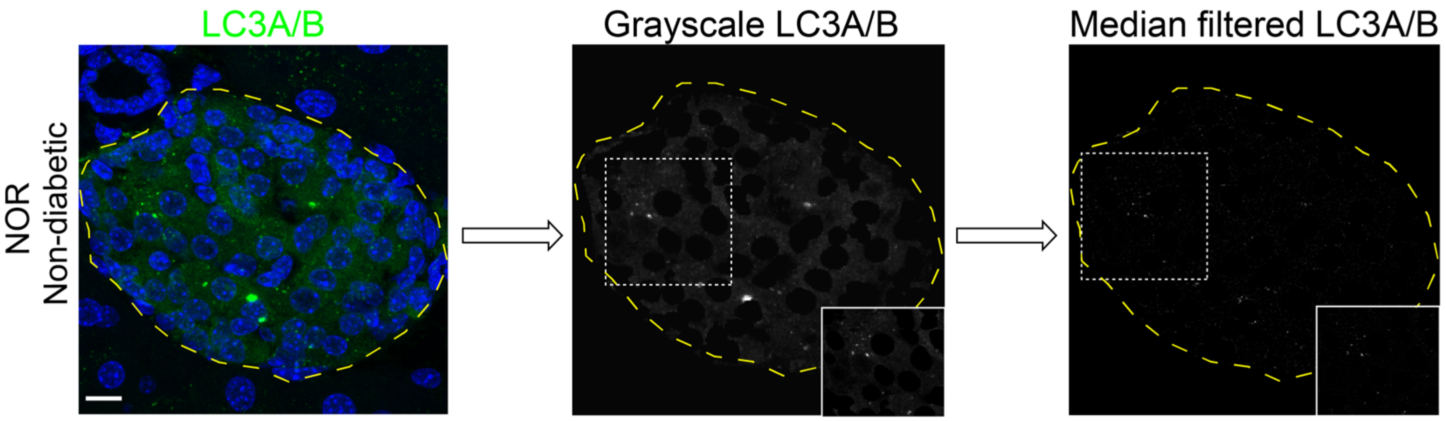

ESM Figure 2. Example of identification of LC3 puncta after applying median filtering using CellProfiler. Scale bar, 10μm.

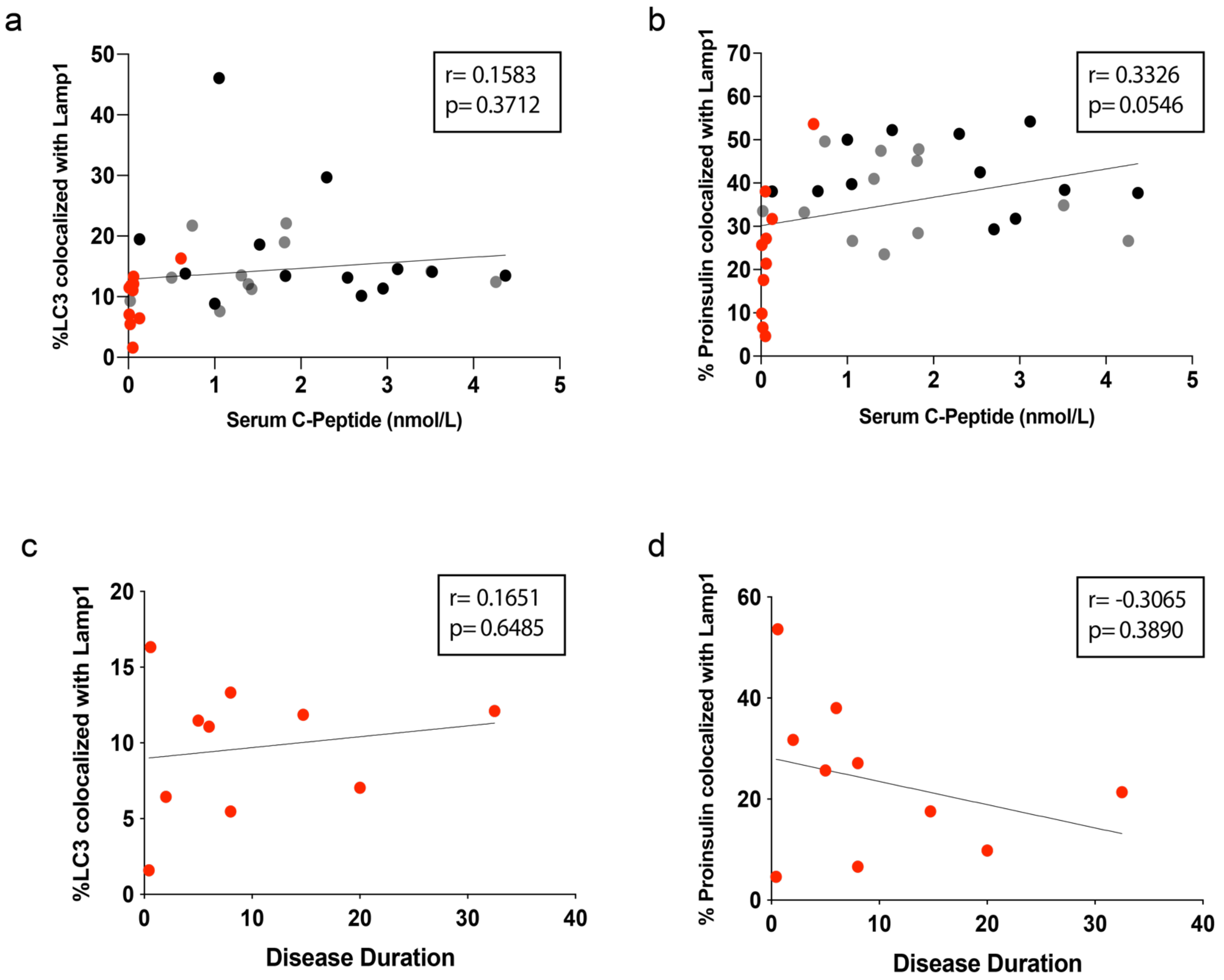

ESM Figure 3. Correlation graphs of autophagy and crinophagy with serum C-peptide levels, and disease duration. Black circles indicate non-diabetic donors, grey circles indicate autoantibody positive donors, and red circles denote donors with type 1 diabetes.

Checklist for reporting human islet preparations used in research

Adapted from Hart NJ, Powers AC (2018) Progress, challenges, and suggestions for using human islets to understand islet biology and human diabetes. Diabetologia https://doi.org/10.1007/s00125-018-4772-2

Islet preparation	1	2	3	4	5	6	7	8[a]
**MANDATORY INFORMATION**								
Unique identifier	6015	6160	6162	6178	6235	6335	6339	6373
Donor age (years)	39	22.1	22.7	24.5	30	18.8	23.3	15.7
Donor sex (M/F)	F	M	M	F	M	M	M	M
Donor BMI (kg/m^2)	32.2	23.9	28.9	27.5	25.4	23.6	25	25
Donor HbA_{1c} or other measure of blood glucose control	N/A	5.2	N/A	5	N/A	5.3	5.3	6.8
Origin/source of islets[b]	nPOD	nPOD	nPOD	nPOD	nPOD	nPOD	nPOD	nPOD
Islet isolation centre	N/A	N/A	N/A	N/A	N/A	N/A	N/A	N/A
Donor history of diabetes? Please select yes/no from drop down list	No	No	No	No	No	No	No	No
**If Yes, complete the next two lines if this information is available**								
Diabetes duration (years)								
Glucose-lowering therapy at time of death[c]								

Islet preparation	9	10	11	12	13	14	15	16[a]
**MANDATORY INFORMATION**								
Unique identifier	6034	6234	6331	6333	6226	6227	6229	6230

Donor age (years)	32	20	27.1	27.1	38	17	31	16
Donor sex (M/F)	F	F	F	F	F	F	F	M
Donor BMI (kg/m^2)	25.2	25.6	24	24.9	27.2	26.4	26.9	19.4
Donor HbA_{1c} or other measure of blood glucose control	N/A	5.8	5.4	4.7	5.3	N/A	5.5	5.3
Origin/source of islets[b]	nPOD	nPOD	nPOD	nPOD	nPOD	nPOD	nPOD	nPOD
Islet isolation centre	N/A	N/A	N/A	N/A	N/A	N/A	N/A	N/A
Donor history of diabetes? Please select yes/no from drop down list	No	No	No	No	No	No	No	No
**If Yes, complete the next two lines if this information is available**								
Diabetes duration (years)								
Glucose-lowering therapy at time of death[c]								

**Islet preparation**	**17**	**18**	**19**	**20**	**21**	**22**	**23**	**24[a]**
**MANDATORY INFORMATION**								
Unique identifier	6232	6147	6167	6151	6170	6181	6301	6310
Donor age (years)	14	23.8	37	30	34.5	31.9	26	28
Donor sex (M/F)	F	F	M	M	F	M	M	F
Donor BMI (kg/m^2)	20.83	32.9	26.3	24.2	36.9	21.9	32.1	22.4
Donor HbA_{1c} or other measure of blood glucose control	N/A	5.2	N/A	N/A	6.9	N/A	5.5	N/A
Origin/source of islets[b]	nPOD	nPOD	nPOD	nPOD	nPOD	nPOD	nPOD	nPOD

Islet isolation centre	N/A	N/A	N/A	N/A	N/A	N/A	N/A	N/A
Donor history of diabetes? Please select yes/no from drop down list	No	No	No	No	No	No	No	No
**If Yes, complete the next two lines if this information is available**								
Diabetes duration (years)								
Glucose-lowering therapy at time of death[c]								

**Islet preparation**	**25**	**26**	**27**	**28**	**29**	**30**	**31**	**32[a]**
**MANDATORY INFORMATION**								
Unique identifier	6314	6397	6400	6450	6483	6388	6303	6197
Donor age (years)	21	21.16	25.15	22	30.46	25.2	22	22
Donor sex (M/F)	M	F	M	F	F	F	M	M
Donor BMI (kg/m^2)	23.8	29.6	22.2	24.4	20.8	26	31.9	28.2
Donor HbA_{1c} or other measure of blood glucose control	N/A	6	5.5	5.7	5.5	5.7	5.4	5.5
Origin/source of islets[b]	nPOD	nPOD	nPOD	nPOD	nPOD	nPOD	nPOD	nPOD
Islet isolation centre	N/A	N/A	N/A	N/A	N/A	N/A	N/A	N/A
Donor history of diabetes? Please select yes/no from drop down list	No	No	No	No	No	No	No	No
**If Yes, complete the next two lines if this information is available**								
Diabetes duration (years)								

Glucose-lowering therapy at time of death[c]								

Islet preparation	33	34	35	36	37	38	39	40[a]
**MANDATORY INFORMATION**								
Unique identifier	6156	6046	6302	6306	6325	6328	6367	6405
Donor age (years)	40	18.8	38.5	19	20	39	24	29.1
Donor sex (M/F)	M	F	M	M	F	M	M	F
Donor BMI (kg/m^2)	19.8	25.2	20.5	24.5	31.2	24	25.7	42.5
Donor HbA_{1c} or other measure of blood glucose control	N/A	N/A	8.2	10.1	N/A	8.7	8.8	7
Origin/source of islets[b]	nPOD	nPOD	nPOD	nPOD	nPOD	nPOD	nPOD	nPOD
Islet isolation centre	N/A	N/A	N/A	N/A	N/A	N/A	N/A	N/A
Donor history of diabetes? Please select yes/no from drop down list	No	Yes	Yes	Yes	Yes	Yes	Yes	Yes
**If Yes, complete the next two lines if this information is available**								
Diabetes duration (years)		8	32.5	5	6	20	2	0.6
Glucose-lowering therapy at time of death[c]			insulin	insulin	insulin	insulin	insulin	insulin

Islet preparation	41	42	43					
**MANDATORY INFORMATION**								
Unique identifier	6414	6435	6477					

Donor age (years)	23.1	24.75	19.87						
Donor sex (M/F)	M	F	F						
Donor BMI (kg/m²)	28.4	26.9	25.3						
Donor HbA_{1c} or other measure of blood glucose control	14	11.6	10.9						
Origin/source of islets[b]	nPOD	nPOD	nPOD						
Islet isolation centre	N/A	N/A	N/A						
Donor history of diabetes? Please select yes/no from drop down list	Yes	Yes	Yes						
**If Yes, complete the next two lines if this information is available**									
Diabetes duration (years)	0.43	14.75	8						
Glucose-lowering therapy at time of death[c]	Lantus and Novolog	insulin	insulin						
